# Taking a Step Back: Insights into the Mechanisms Regulating Gut Epithelial Dedifferentiation

**DOI:** 10.3390/ijms22137043

**Published:** 2021-06-30

**Authors:** Shaida Ouladan, Alex Gregorieff

**Affiliations:** 1 Department of Pathology, McGill University, Montréal, QC H3A 2B4, Canada; shaida.ouladan@mail.mcgill.ca; 2 McGill Regenerative Medicine Network, Montréal, QC H3A 1A3, Canada; 3 Cancer Research Program, Research Institute of the McGill University Health Centre, Montréal, QC H4A 3J1, Canada

**Keywords:** intestinal stem cells, fetal reprogramming, dedifferentiation, lineage tracing, organoids, Hippo signaling, Wnt signaling

## Abstract

Despite the environmental constraints imposed upon the intestinal epithelium, this tissue must perform essential functions such as nutrient absorption and hormonal regulation, while also acting as a critical barrier to the outside world. These functions depend on a variety of specialized cell types that are constantly renewed by a rapidly proliferating population of intestinal stem cells (ISCs) residing at the base of the crypts of Lieberkühn. The niche components and signals regulating crypt morphogenesis and maintenance of homeostatic ISCs have been intensely studied over the last decades. Increasingly, however, researchers are turning their attention to unraveling the mechanisms driving gut epithelial regeneration due to physical damage or infection. It is now well established that injury to the gut barrier triggers major cell fate changes, demonstrating the highly plastic nature of the gut epithelium. In particular, lineage tracing and transcriptional profiling experiments have uncovered several injury-induced stem-cell populations and molecular markers of the regenerative state. Despite the progress achieved in recent years, several questions remain unresolved, particularly regarding the mechanisms driving dedifferentiation of the gut epithelium. In this review, we summarize the latest studies, primarily from murine models, that define the regenerative processes governing the gut epithelium and discuss areas that will require more in-depth investigation.

## 1. A Brief Survey of the Cellular Components and Drivers of the Homeostatic Crypt

The basic unit of the intestinal epithelium is the crypt–villus axis. Crypts are created by invagination of the epithelium into the underlying stroma in a process that begins during late organogenesis and is completed postnatally in mice, whereas, in humans, it is completed several weeks prior to birth [1]. Crypts are primarily composed of proliferative progenitor cells committed to various epithelial lineages, as well as a smaller population of bone fide stem cells, appropriately termed crypt base columnar cells (CBCs) or simply intestinal stem cells (ISCs). Additional folding of the small intestinal epithelium into the lumen creates villi that greatly expands the surface area, thereby facilitating nutrient absorption. The large intestine or colon lacks villi but retains crypt structures divided into proliferative and differentiated zones at the crypt bottom and surface, respectively. Based on morphology, one can distinguish five classes of specialized cells, which can be further subdivided into multiple molecular subtypes on the basis of recent single-cell RNA profiling [2,3]. In brief, absorptive enterocytes are the main components of the epithelium and are characterized by their columnar shape and luminal brush border. Goblet and enteroendocrine cells are responsible for mucus and hormone secretion, respectively, and are distributed sporadically along the crypt–villus axis. Paneth cells are located at the base of crypts, and their bactericidal function defends the gut epithelium against a variety of pathogens. The gut epithelium also harbors specialized cell types responsible for modulating immune cell function. Indeed, tuft cells are chemo-sensory cells, which are necessary for sensing luminal content and play important roles during protozoan and parasitic infections [4]. Lastly, overlaying Peyer’s patches, microfold (M) cells facilitate endocytic transport of luminal antigens to intestinal immune cells [5]. Ultimately, all specialized cells emerge from the crypt base fueled by the constant turnover of ISCs and their immediate progeny.

The balance between self-renewal and differentiation in ISCs is largely determined by Wnt, Notch, Egfr/ErbB, and Bmp signaling pathways, which have been extensively reviewed elsewhere [6,7,8,9,10]. Research into ISCs and Wnt signaling has gone hand in hand over the last decades. The transcriptional signature of ISCs under homeostatic conditions is largely composed of Wnt target genes Lgr5, Ascl2, Rnf43, Axin2, etc. [11]. Disruption of Wnt signaling components including the transcriptional regulators Tcf4 and β-catenin in the intestinal epithelium or transgenic inhibition of extracellular Wnt ligands through secreted Dkk1 results in a significant reduction of crypt proliferative activity [12,13,14,15]. On the other hand, ectopic stimulation of the Wnt pathway by transgenic expression of the Wnt agonist R-Spondin-1 or deletion of the tumor suppressor Apc causes hyperproliferation of intestinal crypts [16,17]. Paradoxically, Wnt signals also promote differentiation of Paneth cells [18,19], presumably through induction of transcription factors such as Sox9 and Spdef [20,21], and they also activate EphB-dependent cell sorting of Paneth cells to the crypt bottom [22]. Similar to Wnt signaling, the Notch pathway is another crucial driver of ISC self-renewal. Conditional ablation of Notch receptors or the downstream transcriptional effector Rbp-J results in the transformation of proliferative crypt cells into goblet cells [23,24]. Inversely, overexpression of active Notch receptor in the intestinal epithelium results in a reduction in goblet cells, as well as enteroendocrine (ENC) and Paneth cell differentiation [25,26]. Hence, the Notch pathway promotes ISC self-renewal and contributes to cell fate specification toward the absorptive lineage, at the expense of secretory cell differentiation. 

Another key mitotic signal for crypt cells involves ErbB activation. Several ErbB receptors and ligands are expressed within the ISC niche [27,28], and inactivation of ErbB signaling severely impairs regeneration and tumorigenesis [29,30,31,32], although it is important to point out that homeostatic turnover of the crypt epithelium is unaffected by these single receptor or ligand knockouts, highlighting their functional redundancy in vivo. However, depletion of the common negative regulator of ErbB signaling, the membrane-bound receptor Lrig1, results in a robust expansion of the stem-cell pool [28]. Lastly, BMP ligands (i.e., BMP2 and 4) are expressed in an inverse gradient relative to BMP antagonists (i.e., Grem 1 and 2) along the crypt/villus axis to control differentiation of ISCs [33,34]. Overexpression of the BMP inhibitor, Noggin, results in ectopic crypt formation analogous to patients with juvenile polyposis, the majority of which harbor germline mutations in various components of the BMP signaling pathway [35,36]. Furthermore, conditional deletion of BMP Receptor-1A results in hyperproliferative crypts [37].

Once the essential signaling pathways driving crypt homeostasis were discovered, defining the cellular components of the ISC niche that release these growth factors and morphogens became a topic of intense investigation. Although primarily involved in sterilizing the stem-cell zone through secretion of antimicrobials, Paneth cells serve as sources of Wnt, Notch, and Egfr ligands that help sustain ISCs [27]. Notably, Wnt 3 is specifically secreted by Paneth cells in the mouse intestine [38], and it is required to drive crypt formation in intestinal organoid cultures [39]. Despite their ability to release important growth factors and morphogens and their close proximity to ISCs, loss of Paneth cells does not lead to ablation of ISCs in vivo, due largely to alternative stromal sources of Wnt ligands [40,41]. As a result, several labs employed single-cell RNA sequencing and genetic labeling experiments to profile the extraordinary heterogeneity within mesenchymal lineages of the intestinal lamina propria [42,43,44,45,46,47,48]. Combining marker expression and high-resolution microscopy, several mesenchymal subtypes have been shown to populate the peri-cryptal zone, including a large population of platelet-derived growth factor receptor A (PDGFRa)-expressing fibroblasts, CD81^+^ trophoblasts, and smooth muscle actin (SMA)-positive myofibroblasts and telocytes [49]. Several lines of evidence indicate that these cell types are required for maintenance of crypt proliferation. For instance, organoid reconstitution assays demonstrated that CD81^+^ trophoblasts, which express elevated levels of BMP antagonist Grem1 and Wnt agonist Rspo3 support organoid growth without addition of exogenous growth factors [42]. Furthermore, deletion of Porcn to block Wnt secretion in Foxl1-expresssing telocytes was sufficient to impair crypt homeostasis in the adult [44].

In summary, homeostatic turnover of the gut epithelium relies on a pool of undifferentiated stem cells that populate the base of the crypts. Supported by both Paneth cells and various mesenchymal cell types, ISCs self-renew and give rise to all differentiated cell types that make up the gut epithelium. Under normal conditions, this process is unidirectional, with differentiated cells ultimately dying off and/or shed into the lumen of the gut. In the next part of the review, we see how injury or infection triggers an expansion of the stem-cell pool via a process of dedifferentiation, whereby surviving, partially differentiated cells acquire fetal-like stem-cell characteristics to drive regeneration of the gut epithelium. 

## 2. Fetal-Like Stem Cells Drive Regeneration 

**Figure 1:**
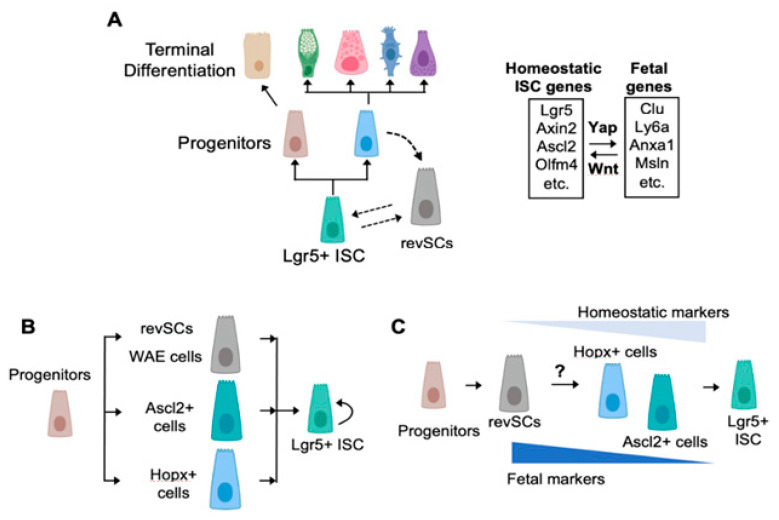
Stem-cell dynamics during intestinal homeostasis and regeneration. (**A**) Under homeostatic conditions, Wnt-driven Lgr5^+^ ISCs maintain epithelial turnover. Following injury, Lgr5^+^ ISCs are lost or reprogrammed, while lineage-committed progenitors adopt a Yap-dependent fetal signature and give rise to de novo Lgr5^+^ ISCs. (**B**) Multiple roads repopulate Lgr5^+^ ISCs. Various regenerative stem-cell populations have been described to date and may represent distinct dedifferentiation processes involved in gut regeneration. RevSCs [55] and wound-associated epithelial cells (WAE cells) [59] express several markers of the pseudostratified fetal gut epithelium and, thus, may represent equivalent cell types. Hopx^+^ cells have been shown to co-express the fetal marker Trop2 (Tacsdtd2), while Ascl2^+^ cells are devoid of fetal markers. (**C**) Single-road model of Lgr5^+^ ISC replenishment. As an alternative scenario, we postulate that, under the influence of Yap signaling, revSCs are the earliest dedifferentiating cell type that may ultimately give rise to Hopx^+^ and/or Ascl2^+^ cells, which acquire progressively more homeostatic features. During this process, fetal and homeostatic stem-cell genes would mark early and late stages of regeneration, respectively. Figure was prepared using Biorender.com.

Several robust injury models based on gamma irradiation or chemical insults have been developed to study regenerative responses in the gut. In these murine models, breaches in the gut epithelium trigger major reorganization of the stem-cell compartment. In particular, ISC markers and Wnt target genes decline following injury and are replaced by a fetal endoderm gene signature (Ly6a, Clu, Anxa1, IL-33, etc.) that is dependent on the Hippo signaling and the transcriptional effector Yap (Figure 1A) [50,51]. Yap and its homolog Taz are tightly regulated by upstream kinases Mst and Lats kinase, which in turn respond to a wide range of extracellular and intracellular cues (see reviews on the subject [52,53]). Intriguingly, Yap-responsive ‘fetal’ genes are normally enriched in the pseudostratified epithelium of the embryonic gut (stage E12–E14 in the mouse), a time point at which Lgr5^+^ ISCs have yet to be fully specified [50,54]. Thus, replenishment of lost ISCs and repair of damaged crypts appear to depend on the temporary reversion of the adult gut epithelium into an embryonic state. Note that this process may not only be restricted to murine intestinal regeneration, as the fetal program is enriched in biopsy material from ulcerative colitis patients [50]. This view is also supported by recent lineage tracing and genetic ablation experiments showing that the Yap-responsive fetal gene, Clusterin (Clu), marks a distinct stem-cell population, termed ‘revival’ stem cells (revSCs), which are required for replenishment of Lgr5^+^ ISCs and crypt regeneration [55] (Figure 1A). In this review, we use the term revSCs to refer to these injury-induced fetal-like stem cells.

At least one important question that emerges from the fetal reprogramming model of intestinal regeneration is whether revSCs are derived from surviving Lgr5^+^ ISCs, their progeny, or both. Our own work and that of others have provided indirect evidence suggesting that Lgr5^+^ ISCs are not a prominent source of regenerating cells following damage [55,56]. Indeed, Yap activation and induction of fetal genes occur throughout the crypt epithelium, suggesting a dependency for Yap in regenerating progenitor populations. Furthermore, lineage tracing studies have shown that Lgr5^+^ cells from irradiated mice contribute little to their de novo replenishment and crypt regeneration. By contrast, genetically labeled progeny were found to be major drivers of ISC recovery. Similarly, numerous studies tracking the fate of tuft cells, Paneth cells, Goblet cells, and enterocytes have shown that lineage committed cells are capable of dedifferentiating into multipotent ISCs during gut regeneration [57]. Together, these results suggest a model whereby lineage-restricted progenitors dedifferentiate into a fetal-like state in response to tissue damage and Yap induction. However, it should be noted that Sato et al. recently questioned the relative contribution of surviving Lgr5^+^ ISCs versus progenitors in driving crypt regeneration [58]. Indeed, contrary to the abovementioned studies, these authors found by genetic labeling that the majority (approximately 72%) of surviving Lgr5^+^ ISCs contributed to the formation of de novo stem cells following irradiation. The underlying reason for this discrepancy is unclear, but the authors also found that surviving Lgr5^+^ ICS are heterogeneous and a fraction of these cells express Yap-dependent fetal genes. Thus, it is plausible that regenerating Lgr5^+^ ISCs may also undergo a Yap-dependent reprograming event, as we originally postulated [51], which temporarily shifts these cells into a slowly cycling revSC state. In conclusion, it is probably safe to suggest that both surviving progenitors and Lgr5^+^ ISCs contribute to crypt regeneration to varying degrees and may both exist in flux between Wnt and Yap-dependent cellular states (Figure 1A).

## 3. How Many Roads Lead to Rome? 

In addition to Hippo-dependent revSCs and surviving Lgr5^+^ ISCs, other regenerative cell types have been described. For instance, Stappenbeck and colleagues used a colonic biopsy injury system to identify wound-associated epithelial (WAE) cells that migrate over the wound bed to re-establish the epithelial barrier [59]. Analogous to revSCs, WAE cells are not proliferative and express markers, which we previously found to be Yap-regulated genes including Cd55, Cldn4, and Dpcr1 [51] (Figure 1B). More recently, the Stappenbeck lab used a lineage tracing approach to demonstrate that epithelial repair following acute DSS treatment depends on Hopx-expressing cells (Figure 1B) [60]. Interestingly, the authors showed that Hopx expression declined in atrophic non-proliferative crypts in the immediate post-injury phase but re-emerged 7 days post DSS treatment in hypertrophic, proliferative crypts lacking Lgr5^+^ cells. It is worth noting that, although Hopx^+^ regenerating crypts expressed the fetal marker Tacstd2, our scRNAseq profile of irradiated crypts [55] found no overlap between Hopx-expressing cells and revSCs. Thus, one may speculate either that Hopx^+^ cells arise independently of revSCs or that revSCs may be transiently induced at earlier stage in the regenerative process and subsequently transition into Hopx^+^ cells (Figure 1B,C). Future experiments designed to compare the kinetics of expression between Hopx and fetal markers such as Clu and Hopx will be particularly informative to determine the sequence of events that are associated with crypt regeneration. 

Using mice harboring the diphtheria toxin (DT) receptor in the Lgr5 locus (Lgr5–DTR mice), Shivdasani and colleagues studied the consequences of ablating Lgr5^+^ ISCs [56]. Previous work by Tian et al. showed that crypt architecture and proliferation remain unimpaired upon DT treatment of Lgr5–DTR mice [61]. The Shivdasani lab utilized this model to show that replenishment of Lgr5^+^ ISCs following cessation of DT treatment depends on and is preceded by upregulation of Ascl2 in regenerating crypt cells above the stem-cell compartment. These data suggest that dedifferentiating progenitors upregulate Ascl2 to regenerate ISCs (Figure 1B). Transcriptional profiling of Ascl2^+^ regenerating cells showed that these cells lack a revSC signature, which is consistent with our scRNAseq data [55] showing that revSCs display low levels of homeostatic ISC markers such as Ascl2 [62,63]. On the basis of these results, the authors concluded that reacquisition of homeostatic markers of stemness depends on induction of Ascl2 and is independent of a Yap-dependent fetal reprogramming event. However, as noted above in the context of Hopx^+^ crypts, an alternative scenario may be proposed whereby Ascl2 induction represents a comparatively late event in the dedifferentiation process which follows the initial and transient Yap-mediated fetal reversion of regenerating crypt cells (see Figure 1C for more details). Once again, more refined studies will be required to map the real-time trajectories of regenerating cells as they replenish homeostatic Lgr5^+^ ISCs.

## 4. Regeneration in a Dish

Intestinal organoids mirror, in many respects, the behavior of the regenerating crypt epithelium in vivo. Seeding of organoids from freshly isolated crypts or single ISCs activates Yap-dependent fetal genes. This was comprehensively demonstrated by Liberali and colleagues, who visualized in real time and transcriptionally profiled developing organoids derived from single Lgr5^+^ ISCs [64]. These authors also presented data suggesting a primary role for Yap in establishing the ISC niche through induction of Notch/DLL1 lateral inhibition. While preparing this review, Tallapragada et al. also reported that crypt fission in established organoid cultures is proceeded by ion channel-dependent inflation and contraction dynamics characterized by transient induction of revSC genes [65]. The authors showed that, under normal growth conditions, Lgr5^+^ stem-cell zones within organoids transiently expand spherically with individual cells adopting a stretched morphology reminiscent of squamous epithelium. The spherical and stretched appearance of organoids is analogous to fetal organoids, which, unlike adult organoids, grow independently of Lgr5^+^ ISCs, adopt a spheroidal morphology, and express high levels of Yap-responsive genes [54,66]. scRNAseq confirmed that stretched epithelial cells lack ISC markers and are enriched for revSC genes including Clu, Basp1, and Anxa1. Most importantly, pharmacologically blocking organoid swelling suppressed fission of Lgr5^+^ stem-cell zones, perhaps implying that stretched cells in developing organoids give rise to new Lgr5^+^ stem cells, analogous to revSC-dependent formation of de novo stem cells during in vivo crypt regeneration.

Lastly, the Stappenbeck group developed a novel self-organizing two-dimensional (2D) epithelial monolayer system to study gut regeneration. When grown in an air–liquid interface, monolayers of colonic organoids fully mature into various cell types, while still maintaining a subpopulation of proliferative stem cells [60]. By contrast, submerged monolayers in the media adopted a regenerative signature including several fetal markers (e.g., Clu, Ly6a, and Tacstd2). Using this system, the authors also demonstrated that the conversion between homeostatic and regenerative states is controlled by oxygen levels, Hif1a signaling, and an ER stress response. Whether these findings are related to Hippo signaling is unclear, but they may suggest that Yap activity is controlled by local changes in oxygen availability.

## 5. Impact of the Microbiota and Infection on Stem-Cell Behavior and Regeneration 

In addition to the suite of injury models described above, several groups are now examining how infectious agents modulate intestinal stem cell behavior. Microbial products are detected by epithelial cells in part through various pattern recognition receptors such as Toll-like receptors (TLRs). Indeed, activation of TLRs by commensal microflora is necessary for the protection against gut injury [67]. The TLR4 ligand, LPS, was found to suppress crypt proliferation through RIPK3-mediated necroptosis and concurrently enhance cell differentiation [68]. In *Drosophila*, Toll like receptor signaling has been shown to crosstalk with the Hippo pathway, but a direct link between these two signaling modules has not been demonstrated yet in mammalian cells [69]. The nucleotide-binding oligomerization domain-containing protein 2 (NOD2), another innate immune receptor, promotes survival of Lgr5^+^ ISCs and repair of the murine crypt epithelium following genotoxic stress through recognition of the muramyl dipeptide (MDP), a peptidoglycan motif common to all bacteria [70,71]. Bacteria also function as important metabolic factories that breakdown indigestible products of the host diet. One such byproduct of microbial metabolism is butyrate, which derives from dietary fiber in the colon. As residents of the crypt bottom, ISCs are normally shielded from butyrate, which is metabolized by surface colonocytes. However, in organisms that lack intestinal crypts (i.e., zebrafish) or upon crypt erosion by DSS administration in mice, ISC proliferation and colonic regeneration is suppressed by butyrate [72]. Another avenue for microbial-dependent regulation of stem cells involves secondary bile acids [73,74]. Unprocessed primary bile acids generated in the liver are metabolized by gut microbiota into secondary bile acids that serve as signaling molecules through interaction with their cognate receptors, which are expressed in Lgr5^+^ ISCs. For instance, loss of the bile acid G-protein-coupled bile acid receptor, TGR5, impairs Lgr5^+^ ISC homeostatic self-renewal and fate specification, as well as regeneration following DSS treatment [73]. Interestingly, TGR5 promotes a regenerative program by activating a Src–Yap axis that leads to fetal gene induction. Lastly, other metabolites may act on niche cells rather than directly stimulating stem cells to support intestinal regeneration. For instance, bacterial-derived lactate stimulates Gpr81-dependent Wnt3 secretion from Paneth cells and stromal cells to promote crypt regeneration in the mouse intestine [75].

Viral infections have also been linked to expansion of ISCs. Specifically, rotavirus, a small intestinal pathogen that infects villus enterocytes leading to diarrhea and vomiting, causes enhanced proliferation of ISCs and turnover of their progeny [76]. In this case, however, expansion of the ISC compartment rests on increased epithelial secretion of Wnt ligands and not progenitor cell-driven dedifferentiation and replenishment of ISCs. The best example of the latter comes from the world of parasitology where intestinal helminth infection has long been associated with increased epithelial turnover [77]. More recently, Klein and colleagues demonstrated that crypt epithelial cells overlaying a submucosal granuloma formed by the rodent dwelling parasitic roundworm *Heligmosomoides polygyrus bakeri* (Hpb) undergo a fetal reversion including suppression of homeostatic ISC markers and emergence of a Sca-1^+^ stem-cell population [78]. Interestingly, the authors demonstrated that fetal-like reprogramming required leukocyte-derived IFNγ signals. An important question arising from these studies is whether microbe or parasite-derived signals act in a cell-autonomous manner to regulate the ISC niche.

## 6. Fibro-Inflammatory Signals Drive Fetal Reprogramming

Reparative processes are typically associated with an influx of immune cells, which release a cocktail of proinflammatory and fibrotic factors that remodel the tissue microenvironment. One of the consequences of this process is increased mechanical stress imposed upon epithelial cells. Mechanotransduction pathways are one of the most prominent means of regulating Yap transcriptional activity [79]. Indeed, Jensen and colleagues showed that collagen deposition triggers an Integrin/FAK/Src axis that promotes Yap-mediated repair of the colonic epithelium and fetal reversion [50]. Inflammatory cytokines are additional regulators of Yap transcriptional activity in the gut. Karin and colleagues demonstrated that IL-6 signals through gp130, which in turn activates Yap in a STAT3-independent fashion [80]. Similarly, a more recent study showed that type 3 innate lymphoid cells (ILC3s) promote Yap activity and crypt regeneration in response to methotrexate treatment via a gp130/Src-dependent mechanism [81]. Lastly, prostaglandin E2 (PGE2), an inflammatory lipid mediator secreted by fibroblasts and macrophages in the gut, has been shown by multiple groups to promote mucosal repair and fetal reprogramming [82,83,84]. Since PGE2 is a known activator of adenylate cyclase and cAMP formation, which in turn regulates GSK3beta, PGE2 was initially suggested to activate β-catenin-dependent transcriptional activity [85]. However, more recent studies have emphasized the ability of PGE2 to induce Yap dephosphorylation and directly stimulate Yap activity [82]. 

Other immune-derived signals may promote epithelial plasticity independently of Hippo signaling. As mentioned above, epithelial specific loss of IFNγR1 prevented induction of the fetal marker Sca-1 in response to helminth infection [78]. In the gastric epithelium, type II innate lymphoid cells (ILC2s) are required for induction of chief cell metaplasia. Infection with *Helicobacter pylori* or chemical injury leads to induction of metaplastic cell lineages, notably the appearance of abnormal mucus-producing cells at the base of the gastric glands, known as spasmolytic polypeptide/trefoil factor 2-expressing metaplasia (SPEM) [82]. Work by the Goldenring and Pizarro labs showed that SPEM induction is dependent on ILC2 production of IL-13 in response to IL-33 signals [86,87,88]. Interestingly, this type of immune circuit is particularly active during enteric helminth infections, suggesting an important role for ILC2 in regulation of epithelial plasticity throughout the gastrointestinal tract.

## 7. Cell-Autonomous Regulation of Epithelial Plasticity

Although several upstream regulators of Hippo signaling in the gut have emerged, the downstream Yap responsive genes that mediate injury-induced reprogramming of the gut epithelium and crypt regeneration remain less well understood. We have shown in organoid cultures that the Yap-responsive gene and Egfr ligand, Epiregulin (Ereg), can rescue crypt formation in Yap-deficient organoids implying an important role for Ereg in mediating crypt outgrowth [51]. Whether Egfr stimulation acts primarily as a mitotic signal or influences cell fate decisions is unclear. Work in other fields has pointed to potentially relevant Yap-dependent processes. Early studies in the liver demonstrated that ectopic Yap activation promotes hepatocyte dedifferentiation through Notch signaling [89]. Furthermore, Picollo and colleagues demonstrated that Yap/Taz-mediated autophagic flux regulated dedifferentiation and acquisition of self-renewing properties in pancreatic and mammary organoid cultures [90]. Specifically, the authors found that Yap/Taz drove expression of Armus, an RAB7 GAP required for autophagosome turnover. It is interesting to note that autophagy has been associated with trans- or dedifferentiation events in other gastrointestinal tissues. Mills and colleagues found that the development of metaplastic lineages in the gastric epithelium or the pancreas proceeds via a series of checkpoints regulated by the nutrient sensor mTORC1 [91]. Immediately following injury, mTORC1 activity declines allowing for an increase in lysosomal and autophagic activity. At later stages, mTORC1 is reactivated to suppress autophagy and initiate the S phase of mitosis. Initial suppression of mTORC1 activity is mediated by the injury-induced scaffolding protein, DDIT4, as well as p53 [92]. Lastly, reactivation of mTORC1 is made possible by the gradual decline in DDIT4 and accumulation of IFRD1, a known repressor of p53. Given that Yap crosstalks with mTOR signaling at multiple levels [93], one may speculate that Yap induction in cell reprogramming events may play an additional role in reactivation of mTORC1 activity allowing for cell-cycle progression and tissue repair. 

Recent findings also indicate that the inner workings of epithelial plasticity in the gut are dependent on synthesis of retinoic acid, a vitamin A liposolubule derivative and well- known regulator of cell fate and growth in various developmental processes [94]. By developing a comprehensive image-based screen of intestinal organoids aimed at testing a library of 2789 compounds, Liberali and colleagues identified a wide range of gene networks regulating cell-fate transitions. In particular, the authors found that inhibition of retinoid X receptor (RXR)-α prevented enterocyte differentiation and forced organoids to maintain a Yap-dependent gene signature. Conversely, treatment with an RXR agonist, all-*trans*-retinoic acid (atRA) or retinoic acid (9*cis*-RA), led to Yap cytoplasmic localization and increased enterocyte differentiation. Whether the effects of RXR are mediated directly through regulation of Yap remains unresolved but nevertheless point to a key role for retinol metabolism in maintaining the balance of cell types between enterocytes and undifferentiated progenitors. 

The epigenetic landscape is another well-established determinant of stem-cell fate. Generally speaking, cellular differentiation requires various classes of transcriptional factors that initially displace nucleosomes at specific loci and reorient flanking nucleosomes by recruitment of ATP-dependent chromatin remodeling factors and histone-modifying enzymes [95]. In the homeostatic gut epithelium, however, the landscape of chromatin accessibility and histone modifications between progenitors (both enterocyte and secretory lineages) and ISCs is generally similar, with the exception of secretory cells, which display distinct chromatin accessibility profiles [96,97,98,99]. Interestingly, following loss of ISCs, secretory cell specific enhancers rapidly adopt a closed conformation as they dedifferentiate into de novo ISCs. Against this backdrop, recent organoid-based studies indicate that specific epigenetic regulators may play an important role in fetal reprogramming. A compound screen performed by the Oudhoff lab targeting several methyltransferases and demethylases identified an inhibitor of lysine-specific demethylase 1 (LSD1) as a potent repressor of Paneth cell differentiation [100]. Further investigation revealed that, despite losing Paneth cell-derived niche signals, treated organoids displayed enhanced Lgr5^+^ ISCs and fetal gene expression in a Yap/Taz-independent fashion. Consistent with the role of LSD1 as a transcription corepressor through demethylation of lysine 4 on histone H3 (H3K4), chromatin immunoprecipitation sequencing (ChIP-seq) in wild-type and LSD1-deficient cells showed that LSD1 controls H3K4 methylation of fetal-like gene loci. Another clue that epigenetic regulators are important mediators of fetal reprograming during gut regeneration came from the recent work of Deng and colleagues [101]. Indeed, these authors showed that treatment of organoids with a cocktail of chemical inhibitors and growth factors (i.e., LDN193189, GSK-3 inhibitor XV, pexmetinib, VPA, EPZ6438, EGF, R-Spondin 1, and bFGF) caused hyperplastic growth and induction of a revSC signature. Notably, removal of valproic acid (VPA), a histone deacetylase inhibitor, and EPZ6438, a selective inhibitor of the lysine methyltranferase EZH2, led to reduced expression of fetal-associated genes. Furthermore, in vitro and in vivo treatment with VPA and EPZ6438 was sufficient to enhance crypt regeneration in a Yap-dependent matter. Together these organoid-based studies reveal novel mechanisms regulating regenerative processes in the gut epithelium and pave the way to exploring pharmacological approaches that may have therapeutic value. 

## 8. Concluding Remarks

**Figure 2:**
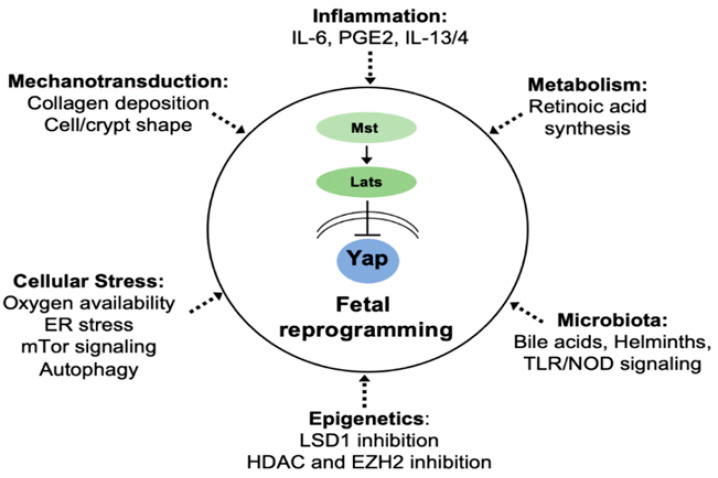
Regulation of fetal reprogramming and dedifferentiation in gastrointestinal tissues. Several processes including collagen deposition, IL-6, prostaglandin E2 (PGE2), retinoic acid synthesis, and bile acid receptor signaling have been reported to directly regulate Hippo/Yap-dependent fetal gene expression. Other processes mentioned and discussed in the text may promote dedifferentiation via alternative mechanisms. However, further investigation into the mechanisms regulating intestinal plasticity is required.

In summary, epithelial plasticity in the regenerating gastrointestinal tract is regulated at multiple levels (Figure 2). At the forefront of this response is the induction of the Hippo signaling effector Yap, which transiently suppresses Wnt-driven ISCs in favor of a fetal gene signature. Yap activity, induction of fetal genes, and crypt regeneration are regulated by several extra- and intracellular signals including collagen deposition, stimulation by inflammatory cytokines, microbial metabolites, modulation of epigenetic regulator expression, and retinal metabolism to name but a few. New findings in other regenerating tissues also point to nutrient sensors and autophagy as possible mediators of early reprogramming events in the regenerating intestine. Despite significant progress in this field, many questions remain to be resolved. Why do regenerating cells adopt a fetal gene signature in the first place? One may suggest that transient reversion of intestinal stem cells into a fetal state confers a survival advantage following stress or injury. If this is the case, which Yap-responsive genes are essential for this process? Many of the Yap signature genes encode secreted factors (e.g., Clu, Ctgf, IL-33, and Areg) that may function in a paracrine fashion and are not necessarily involved in cell-autonomous activity. Indeed, the primary function of regenerating epithelial cells may be to reorganize the stem-cell niche through recruitment of immune cells and remodeling of mesenchymal lineages. By ‘tilling the soil’ following damage or infection, the regenerating epithelium may help restore sufficient levels of ISCs. In support of this notion, recent evidence suggests that the intestinal stem-cell niche is also highly plastic and responsive to epithelial-derived signals [48,102,103]. Thus, it will come as no surprise that we have only scratched the surface when it comes to our understanding of the intrinsic and extrinsic processes regulating epithelial plasticity and regeneration in the gut, not to mention that we have barely begun to address the similarities and differences driving gut regeneration in humans vs. mice. As we continue to learn more about these mechanisms in the future, it will be important to capitalize on these discoveries by targeting regenerative pathways for the treatment of diverse intestinal diseases.

## Data Availability

Not applicable.
